# Supplementary material for: Tracking Brownian motion in three dimensions and characterization of individual nanoparticles using a fiber-based high-finesse microcavity
Source: Nat Commun. 2021 Nov 4;12:6385. doi: 10.1038/s41467-021-26719-5 (PMC8569196; doi:10.1038/s41467-021-26719-5)
Supplement: Supplementary file 1 — Supplementary Information [file 41467_2021_26719_MOESM1_ESM.pdf]

Supplementary information:
Tracking Brownian motion in three dimensions and characterization of individual nanoparticles using a fiber-based high-finesse microcavity

Larissa Kohler,[1,*] Matthias Mader,[2,3] Christian Kern,[4,5] Martin Wegener,[4,5] and David Hunger[1,6,†]

[1] *Karlsruher Institut für Technologie, Physikalisches Institut, Wolfgang-Gaede-Str. 1, 76131 Karlsruhe, Germany*
[2] *Fakultät für Physik, Ludwig-Maximilians-Universität, Schellingstraße 4, 80799 München, Germany*
[3] *Max-Planck-Institut für Quantenoptik, Hans-Kopfermann-Str. 1, 85748 Garching, Germany*
[4] *Karlsruher Institut für Technologie, Institut für Angewandte Physik, Wolfgang-Gaede-Str. 1, 76131 Karlsruhe, Germany*
[5] *Karlsruher Institut für Technologie, Institut für Nanotechnologie, Hermann-von-Helmholtz-Platz 1, 76344 Eggenstein-Leopoldshafen, Germany*
[6] *Karlsruher Institut für Technologie, Institut für QuantenMaterialien und Technologien , Hermann-von-Helmholtz-Platz 1, 76344 Eggenstein-Leopoldshafen, Germany*

* larissa.kohler@kit.edu
† david.hunger@kit.edu

Supplementary Figure 1: Laser and detection system

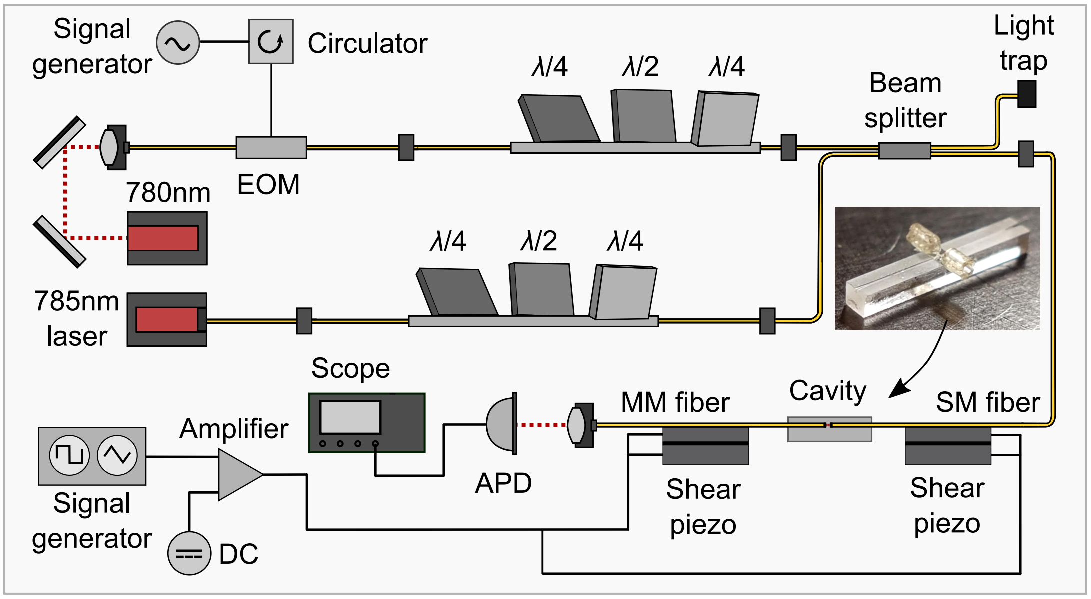

Schematic representation of the measurement setup. The cavity can be probed simultaneously by two different lasers: A tunable grating stabilized external cavity diode laser at the wavelength $\lambda = 780\,\text{nm}$ (TOPTICA DLpro) and a wavelength stabilized laser diode (Thorlabs LP785-SAV50) at $\lambda = 785\,\text{nm}$. The 780 nm laser beam is coupled into a single-mode fiber and its polarization can be changed with a fiber polarization controller ($\lambda/4 - \lambda/2 - \lambda/4$). A 90/10−beamsplitter (Thorlabs TN785R2A2) allows the coupling of both wavelengths into the cavity single-mode (SM) fiber. Without the need of any coupling optics, the light is directly coupled into the cavity. The transmitted light from the cavity is then collected by a multi-mode (MM) fiber, detected with an avalanche photodetector (Thorlabs APD 110A/M) and recorded by an oscilloscope (LeCroy HRO66Zi).

Each cavity fiber is clamped on a piezo-electric actuator. The actuators are operated in opposite directions to change the cavity length. Here we are using a triangular voltage signal for the operation of the actuators, which is generated by a signal generator and amplified (Falco Systems WMA-280).

The laser can be phase-modulated by an electro-optic modulator (EOM, Laser Components NIR-MPX800) to produce sidebands at precisely known frequency separations, which allow us to calibrate the resonance linewidth in frequency units.

Supplementary Figure 2: Fabrication of the first cavity system by direct laser writing

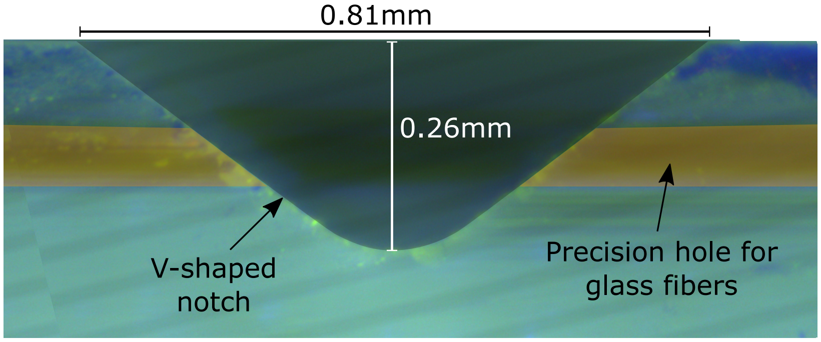

In the following, we describe the fabrication of the cavity system shown in Fig. 1a and b (cavity 1), which comprises a glass ferrule allowing for the precise alignment of the optical fibers forming the cavity and a microfluidic channel, which is oriented perpendicular to the cavity.
In the first step, using an automatic dicing saw (Disco DAD3350), we cut a v-shaped notch into the central part of the glass ferrule, which gives access to the cavity. A corresponding micrograph is shown in the figure above.
In the second step, we fabricate the microfluidic channel using a commercial direct laser writing setup (Photonic Professional GT, Nanoscribe GmbH) with a 25× NA 0.8 objective lens (LCI Plan-Neofluar Imm Corr DIC M27, Carl Zeiss Microscopy GmbH) and the liquid negative-tone photoresist IP-S (Nanoscribe GmbH). The total writing time is about 3.5 h. Due to the large dimensions of the polymer structure, several measures have to be taken: First, we use a galvanometer mirror scanning system to increase the writing speed. Second, we use the so-called dip-in configuration, which means that the objective lens is immersed in the liquid photoresist itself, thereby removing the constraint on the height of the structure imposed by the working distance of the objective lens. Third, instead of polymerizing the whole structure during the actual writing process, we only polymerize an outer shell as well as an internal support scaffold. Following the development of the structure in mrDev 600 (micro resist technology GmbH), the liquid photoresist inside of the structure is cured using a UV flood-exposure.
In the third and last step, in order to ensure that the system is watertight, we apply a hydrophobic coating in a two-step process: First, the structure is conformally coated with a Al_2O_3 layer with a thickness of several ten nanometers using atomic-layer deposition. Second, following a brief air-plasma treatment, the structure is immersed in a 3 mM solution of octadecyltrichlorsilane (CAS 112-04-9) in toluene.

Supplementary Figure 3: Cavity system with a drilled hole for the microfluidic channel

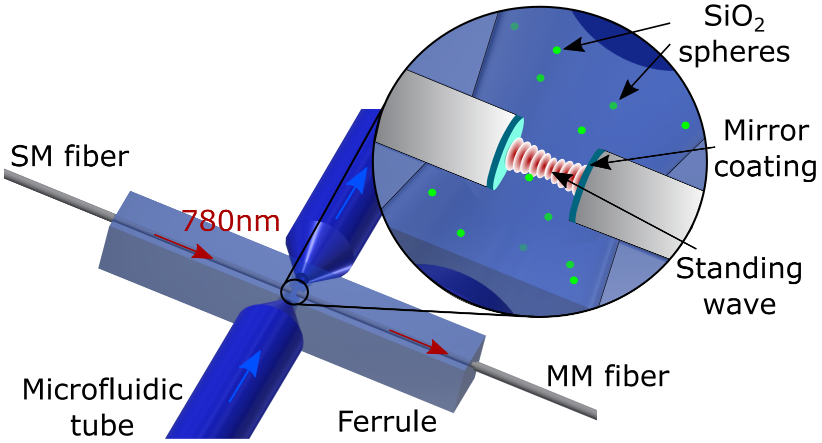

Our experimental data was recorded by two different cavity setups (see Supplementary Note 1). Both setups incorporate a glass ferrule with a precision hole, into which we insert the cavity fibers opposite of each other. In contrast to the photoresist structure of the setup shown in Fig. 1a, here, the microfluidic section consists of a cone-shaped hole, which is drilled into the glass ferrule by a femtosecond laser. The microfluidic hole has a mean outer diameter of 560 µm, a mean inner diameter of 320 µm and intersects the existing precision hole orthogonally in the center of the ferrule. Commercially available microfluidic tubes are directly attached to the microfluidic access from each side. Abbreviations used in the schematic illustration: multi-mode (MM), single-mode (SM) and quartz glass (SiO_2).

Supplementary Figure 4: Extracting time shift and decrease of amplitude from the detection signal

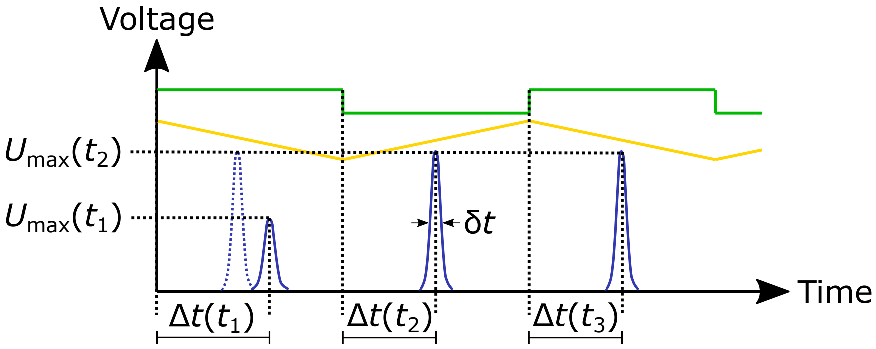

For the measurement of the cavity frequency shift and transmission change, the cavity length is modulated with a triangular voltage driving two shear piezo-electric actuators with a frequency of 3 kHz (yellow). Only when the cavity length hits the resonance condition, the fundamental mode (blue) occurs and can be detected on the photodiode. On each trigger event (green), one sweep action of the piezos is executed. If the cavity is empty (no particle inside the light field), the resonance peak height remains unchanged ($U_{\max} = \text{const.}$), but changes its temporal position Δt due to thermal, acoustic, mechanical and electronic noises mostly at low frequencies. These drifts are then removed by a background subtraction method (see Supplementary Fig. 5). In the schematics, the drift is considered to be constant. The empty cavity condition is shown in the figure at the trigger times t_2 and t_3. If a nanoparticle enters the cavity light field, it produces a decrease of the resonance amplitude and a temporal shift (see trigger event at t_1), which corresponds to a change of the resonance condition of the cavity length. In our experiments, we measure the amplitude value $U_{\max}$, the temporal position Δt and the resonance linewidth δt for each trigger event at time steps of 0.33 ms. Using the sidebands produced by the phase modulation, we convert the measured temporal shift Δt into a frequency shift $\Delta\nu$ (see Supplementary Fig. 5).

Supplementary Figure 5: Data evaluation of nanoparticle transit events

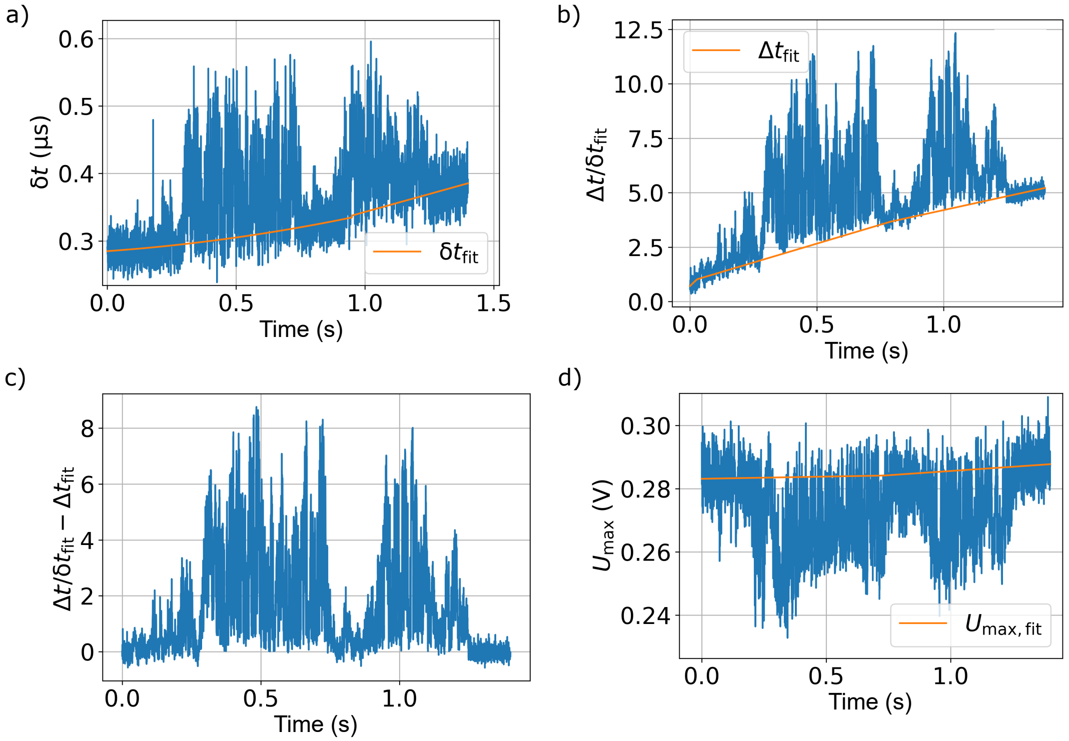

Each nanoparticle transit event produces a change in the resonance linewidth δt, resonance amplitude U_{max} and cavity length depending on the nanoparticle position inside the light field. The cavity length change is measured as a temporal shift Δt (see Supplementary Fig. 4). Supplementary Fig. 5a shows δt over time and a parabola fit (δt_{fit}, orange) to the undisturbed section of the measurement of the linewidth (empty cavity), b) shows the temporal shift in units of cavity temporal linewidths $\Delta t/\delta t_{\mathrm{fit}}$ over time and a parabola fit (Δt_{fit}, orange) to the undisturbed section of the measurement of the temporal linewidths and c) shows the drift corrected shifts $\Delta t/\delta t_{\mathrm{fit}} - \Delta t_{\mathrm{fit}}$. This is converted into a frequency shift by the calibration described above (Supplementary Fig. 1, 4). d) shows U_{max} over time and a parabola fit ($U_{\mathrm{max,fit}}$, orange) to the amplitude values of the undisturbed cavity. The transmission change is defined as

$$\Delta T(t)/T_0 = 100\% \times \left(1 - \frac{U_{\mathrm{max}}(t)}{U_{\mathrm{max,fit}}(t)}\right). \tag{1}$$

Supplementary Figure 6: Time duration of the measured transit events

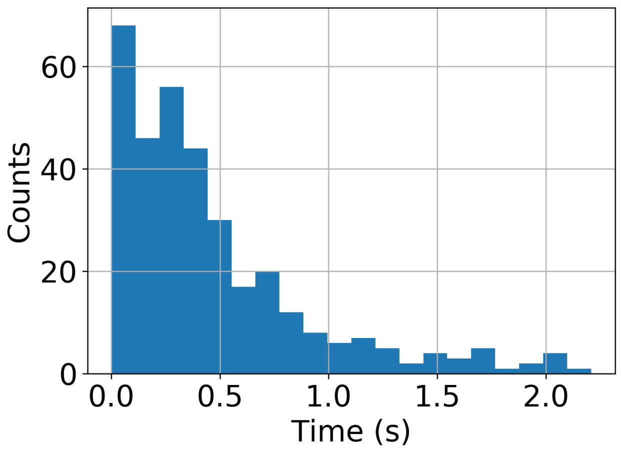

A measured transit event is shown in Supplementary Fig. 5c. From each transit event, the dwell time of the nanoparticle inside the cavity is determined (signal > noise) and all 330 transit events are plotted in the histogram above. The mean dwell time of all transit events is 0.47 s.

Supplementary Figure 7: Determination of the refractive index from the measured decrease of amplitudes

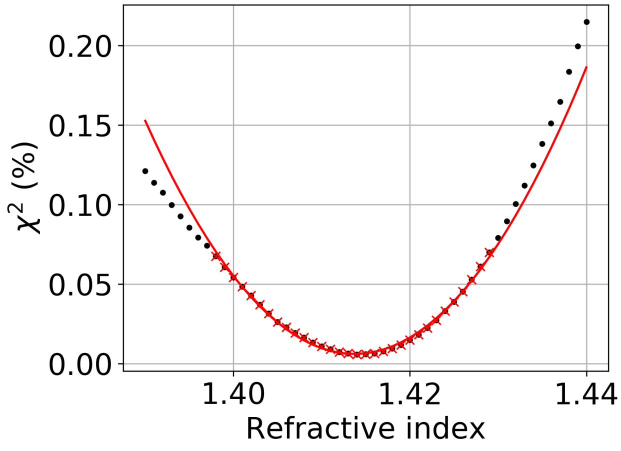

For the measurement of the SiO_2 nanoparticles with $r_{\mathrm{hydr}} = 71.5\,\mathrm{nm}$ (sample A) we used the cavity shown in Fig. 1 with a finesse of $\mathcal{F} = 18400$ and a cavity length of $27.3\,\mu\mathrm{m}$. Due to the fabrication, our cavity mirrors are not perfectly spherical at the center and exhibit different radii of curvatures (ROC) r_{c}:

	$r_{\mathrm{c,x}}$ (µm)	$r_{\mathrm{c,y}}$ (µm)
SM-Fiber	62.4	56.6
MM-Fiber	58.9	131.0

The calculated impact on the transmission change values for the given ROCs are negligible small. Hence, we can use the mean values $\langle r_{\mathrm{c,SM}} \rangle = 59.5\,\mu\mathrm{m}$ and $\langle r_{\mathrm{c,MM}} \rangle = 95.0\,\mu\mathrm{m}$ for the determination of the refractive index n_{NP} from the experimental data in Fig. 2c. In the figure above, the $\chi^2(n_k)$-function is shown. Here we compare different simulations $\Delta T(\nu_{\mathrm{i}}, n_k)$ with fixed particle radius r_{hydr} and different refractive indices n_k (see Supplementary Note 2) with the mean transmission change $\langle \Delta T(\nu_i) \rangle$ at each frequency shift (see black dashed line in Fig. 2c:

$$\chi^2(n_k) = \sum_i \frac{|\Delta T(\nu_i, n_k) - \langle \Delta T(\nu_i) \rangle|^2}{\langle \Delta T(\nu_i) \rangle}. \tag{2}$$

A parabola fit (red) to the center of the minimum (red crosses) gives the value $n_{\mathrm{NP}} = 1.41 \pm 0.01$. The error is deduced from the y-error of the parabola fit.

Supplementary Figure 8: Determination of the refractive index from the measured frequency shifts

With the cavity shown in Supplementary Fig. 3 with a finesse of $\mathcal{F} = 56710$ and a

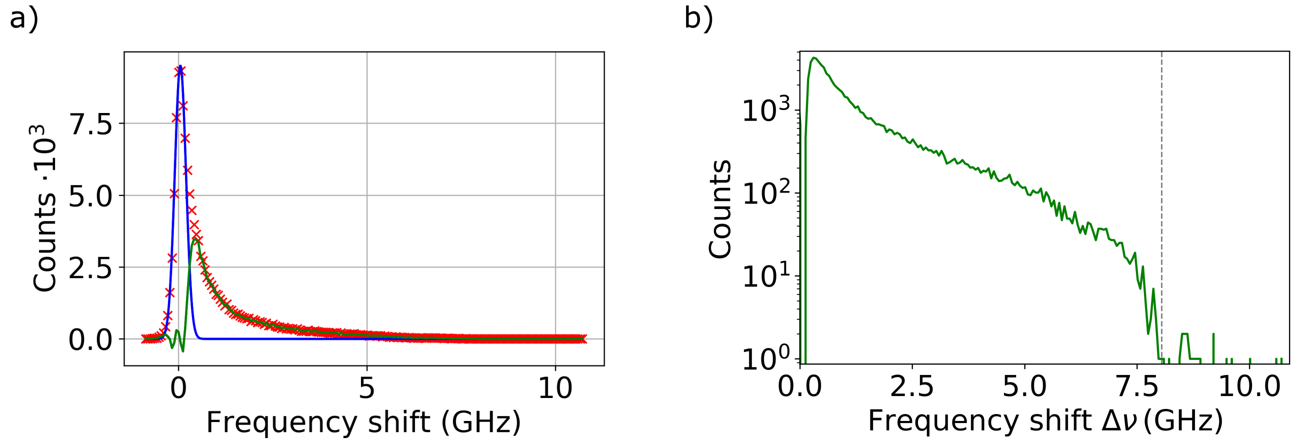

cavity length of 5.4 µm, we measured the frequency shift of the fundamental mode produced by SiO_2 nanoparticles with $r_{\mathrm{hydr}} = (75.3 \pm 9.5)\,\mathrm{nm}$ (sample B). Since we simultaneously measure the position of two higher order cavity modes in addition here, our data acquisition was not able to measure also the transmission change correlated to the frequency shifts, such that we have to extract the refractive index only from the measured frequency shifts. This leads to a larger error for the refractive index and can be avoided in future experiments by always measuring the decrease of amplitude (see Supplementary Fig. 7). In a) we show a histogram of the measured frequency shifts (red crosses) from 59 transit events. The high peak at small frequency shifts arises due to the empty cavity frequency noise of the measurement, and can be fitted by a Gaussian function (blue) and subsequently subtracted from the measured distribution (green). This yields the frequency shifts predominantly produced by the single nanoparticle transits. b) shows the nanoparticle's frequency shifts on a logarithmic scale (green). Here, a characteristic steep decay at 8.05 GHz appears (gray vertical dashed line), which corresponds to the maximum measurable frequency shift for a single nanoparticle located at an antinode of the standing wave cavity light field. In order to determine the refractive index, probability distributions of the theoretical frequency shifts produced by a single nanoparticle with different refractive indices are calculated for the mean mirror curvatures $\langle r_{\mathrm{c,SM}} \rangle = 49.6\,\mu\mathrm{m}$ and $\langle r_{\mathrm{c,MM}} \rangle = 83.5\,\mu\mathrm{m}$ (also see Supplementary Note 3). The maximal occuring frequency shift of 8.05 GHz is expected to stem from the larger nanoparticle diameter with $r_{\mathrm{hydr}} = (75.3 + 9.5) = 84.8\,\mathrm{nm}$. Here we get an effective refractive index of $n_{\mathrm{eff}} = 1.43$. As the size distribution of the

bare nanoparticle only has a standard deviation of $\sigma_r = 1.8\,\mathrm{nm}$, we expect a large systematic error on the effective refractive index due to a wrong assumed size for the larger shift. We estimate the systematic error by assuming a smaller radius $r_{\mathrm{hydr}} = (75.3 + 9.5/2) = 80.05\,\mathrm{nm}$ producing the maximal shift of 8.05 GHz. Here we get $n_{\mathrm{eff}} = 1.45$ and therefore the overall effective refractive index amounts to $n_{\mathrm{eff}} = 1.43 \pm 0.02$. Since the measurement noise and the deviation in the radius of curvature cause much smaller deviations for the refractive index, only the systematic error is considered.

Supplementary Figure 9: SiO_2 spheres with r_{hydr} = 71.5 nm

Sample A was purchased from microParticles GmbH. The nanoparticles are specified to have a mean hydrodynamic radius of $r_{hydr} = 71.5\,\text{nm}$.

a) Sample properties provided by the manufacturer

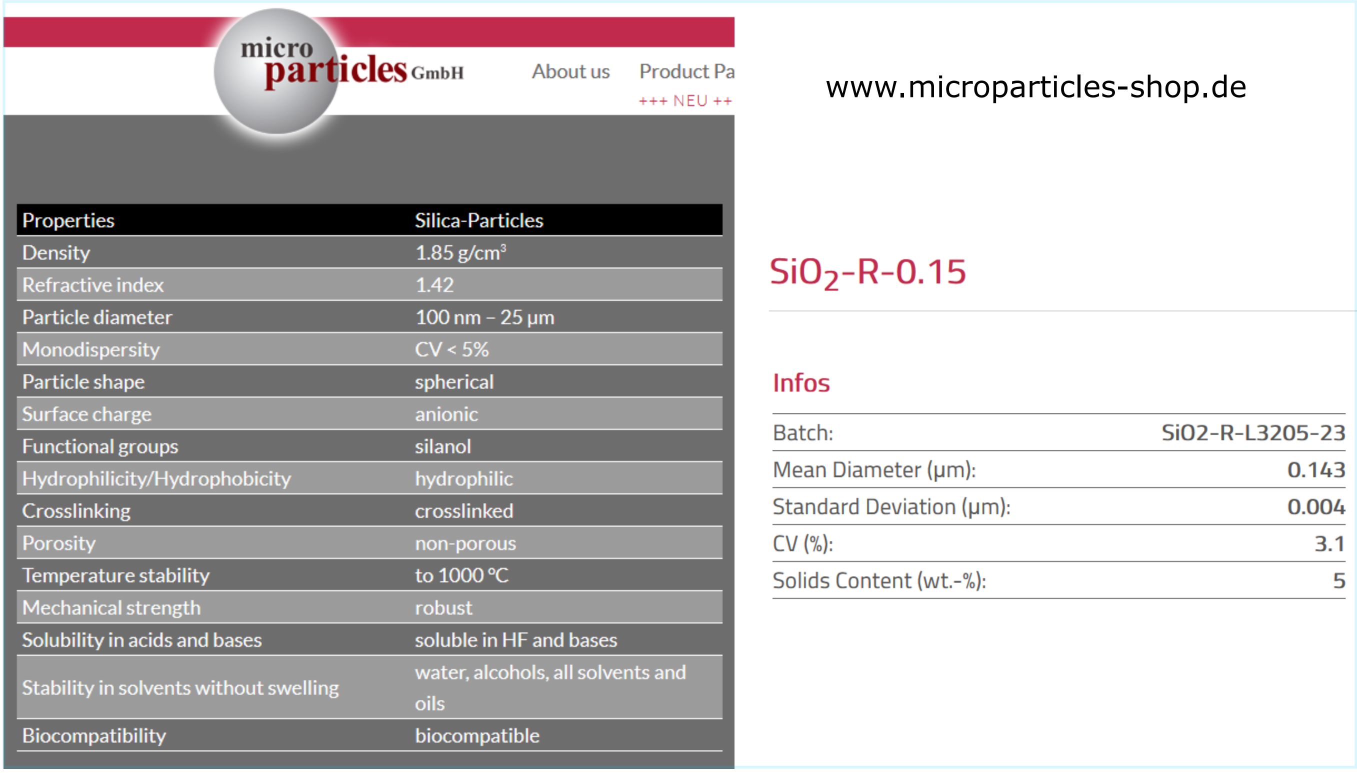

Properties	Silica-Particles
Density	1.85 g/cm³
Refractive index	1.42
Particle diameter	100 nm – 25 µm
Monodispersity	CV < 5%
Particle shape	spherical
Surface charge	anionic
Functional groups	silanol
Hydrophilicity/Hydrophobicity	hydrophilic
Crosslinking	crosslinked
Porosity	non-porous
Temperature stability	to 1000 °C
Mechanical strength	robust
Solubility in acids and bases	soluble in HF and bases
Stability in solvents without swelling	water, alcohols, all solvents and oils
Biocompatibility	biocompatible

Batch:	SiO2-R-L3205-23
Mean Diameter (µm):	0.143
Standard Deviation (µm):	0.004
CV (%):	3.1
Solids Content (wt.-%):	5

The information shown in the figure was taken from the web page www.microparticles-shop.de (June, 2020). We got additional information via email transfer:

The refractive index of the SiO_2 nanoparticles was determined by refractive index matching and their size distribution was determined by dynamic light scattering (DLS) measurements.

b) SEM measurements

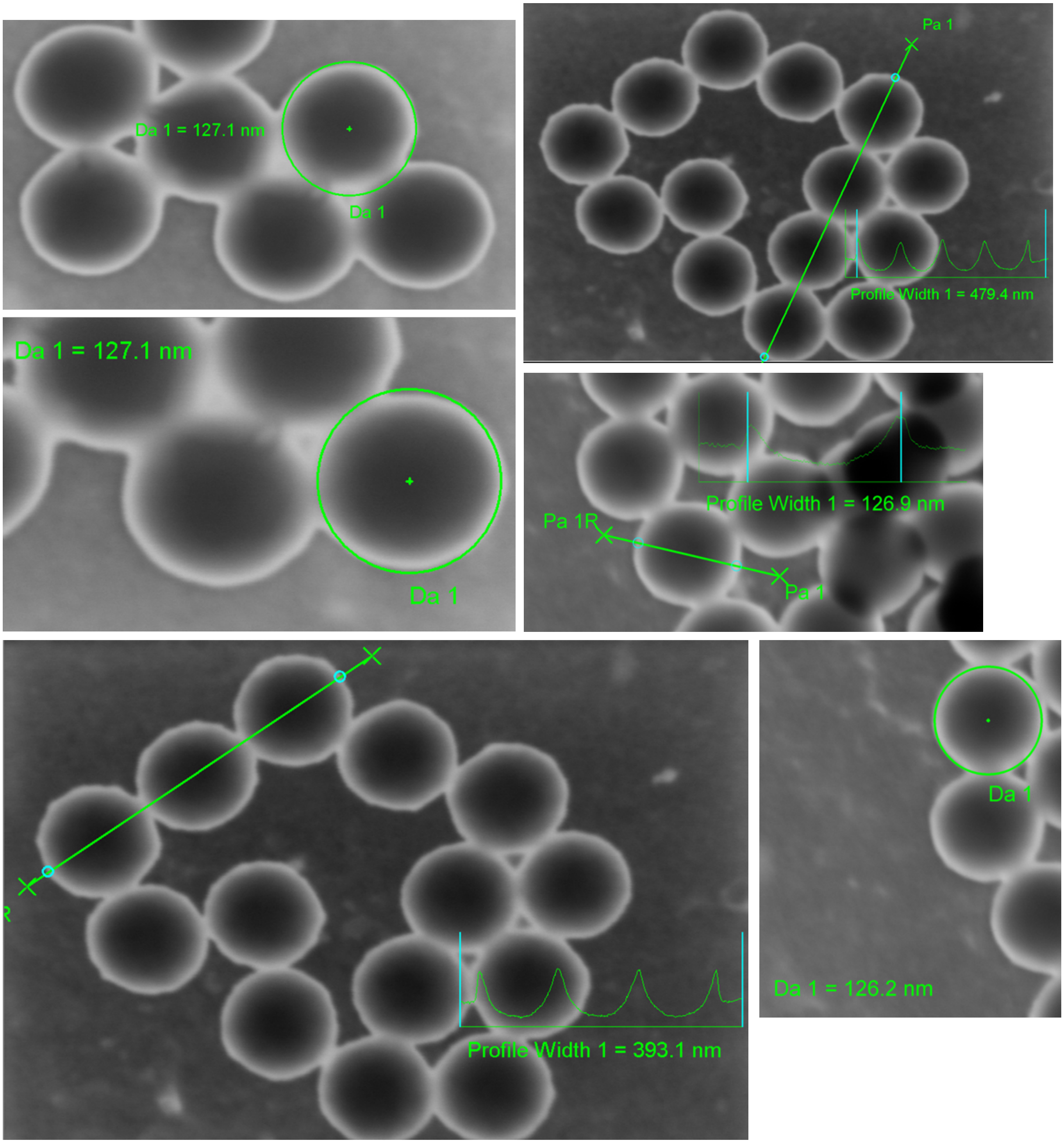

The figure above shows scanning electron microscope (SEM) pictures of the SiO_2 nanoparticles, which are dried on a graphene grating. The measured mean radius is $r_{SiO_2} = 63.5\,\text{nm}$, which is smaller than the hydrodynamic radius of $r_{hydr} = 71.5\,\text{nm}$ measured by the manufacturer via DLS measurements.

Supplementary Figure 10: SiO_2 spheres with $r_{hydr} = 75.3\,nm$

Aug 20, 2019

ALPHA Nanotech

Non-Functionalized Colloidal Silica Nanospheres (120 nm)

Transmission electron micrographs:

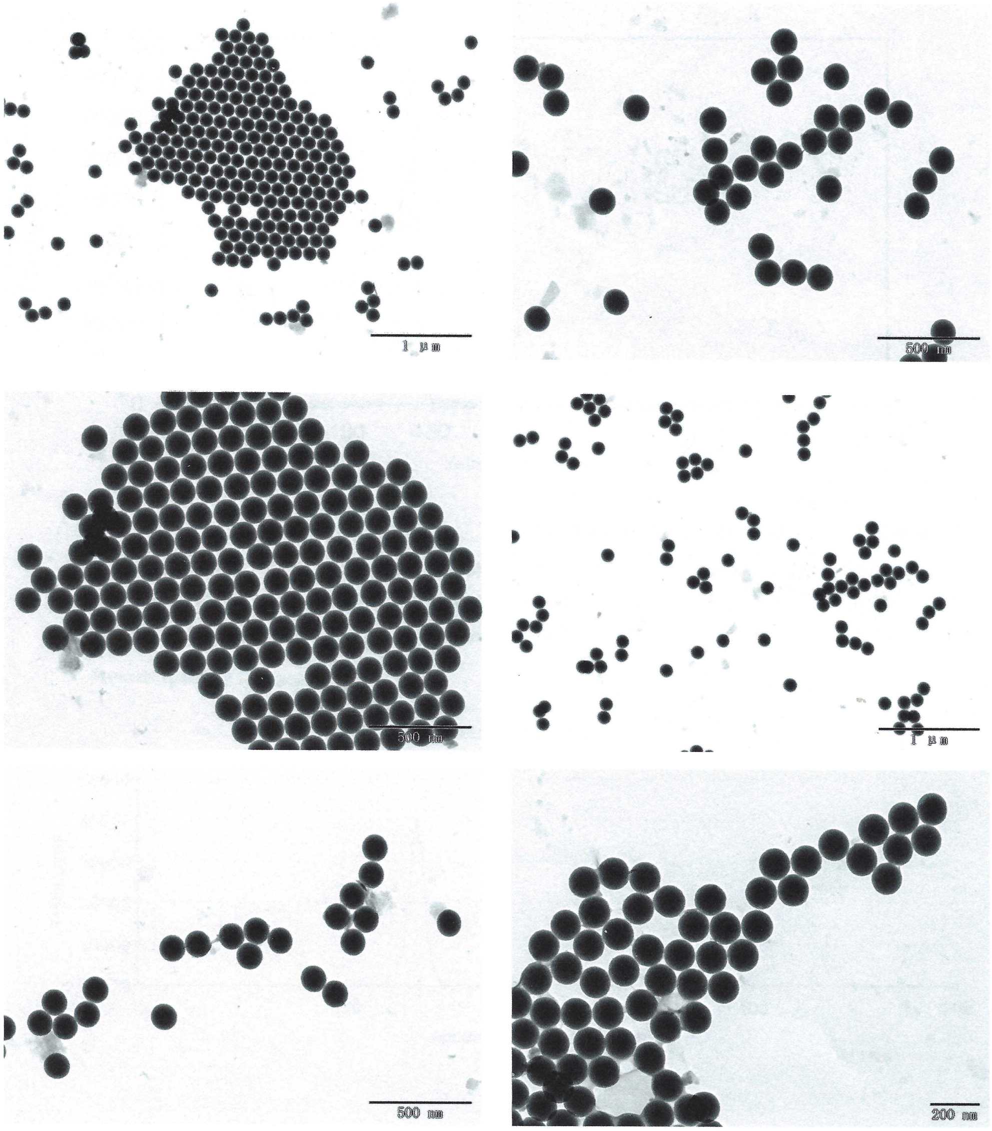

Page 6 of 11

Sample B was purchased from ALPHA nanotech. The nanoparticles were characterized with a transmission electron micrograph (TEM) by the manufacturer, and a physical radius of $r = 60\,\mathrm{nm}$ is specified. The figure above is taken from the

company's data sheet.

Results

			Size (d.n...	% Intensity:	St Dev (d.n...
Z-Average (d.nm):	150,6	Peak 1:	156,7	100,0	34,22
PdI:	0,016	Peak 2:	0,000	0,0	0,000
Intercept:	0,963	Peak 3:	0,000	0,0	0,000
Result quality	Good				

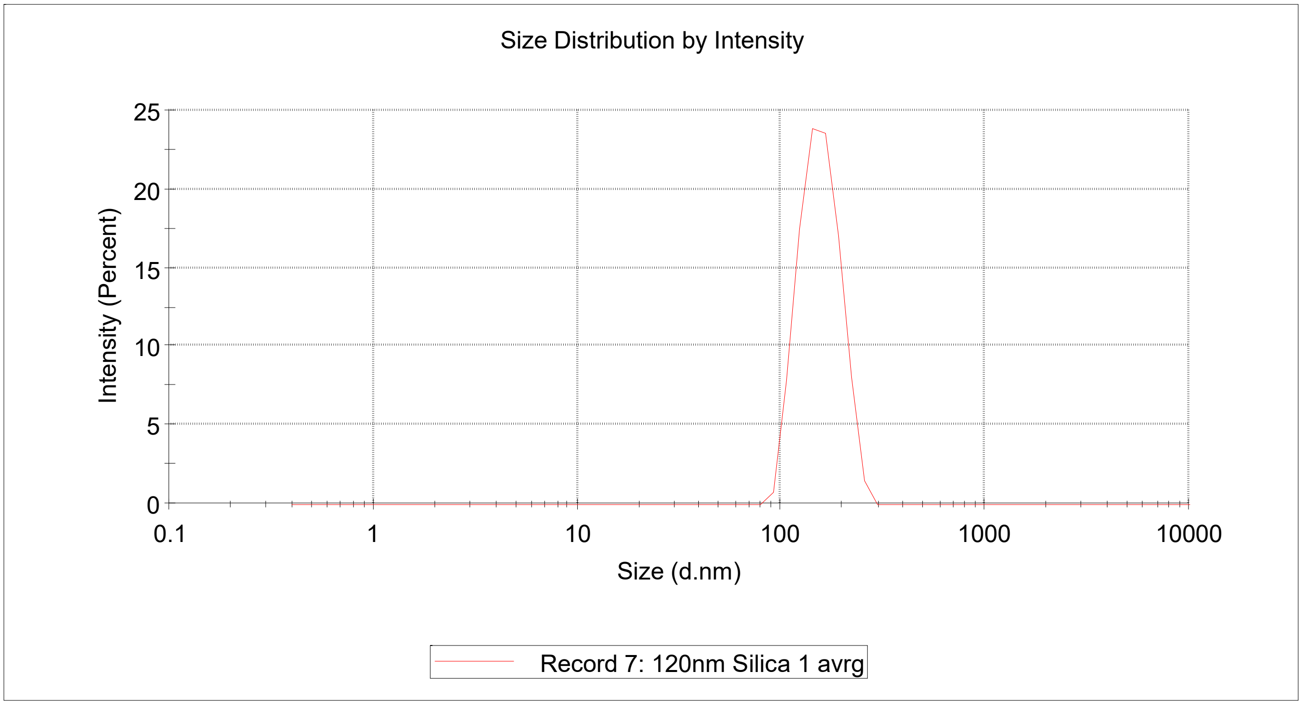

To independently determine the hydrodynamic radius, we have performed DLS measurements of sample B. The z-average yields a hydrodynamic radius of $r_{\mathrm{hydr}} = 75.3\,\mathrm{nm}$ with a standard deviation $\sigma_r = \sqrt{PdI} \cdot r_{\mathrm{hydr}} = 9.5\,\mathrm{nm}$.
Via email transfer we got from ALPHA nanotech the information that they didn't measure the refractive index of the glass nanospheres, but "as there is no impurity added into the silica nanospheres, the silica nanospheres should have the same physical parameters as silica materials". Accordingly, we assume the refractive index of the bulk silica nanospheres to be $n_{\mathrm{SiO2}} = 1.4537$ at $780\,\mathrm{nm}$.

Supplementary Figure 11: Hydration shell

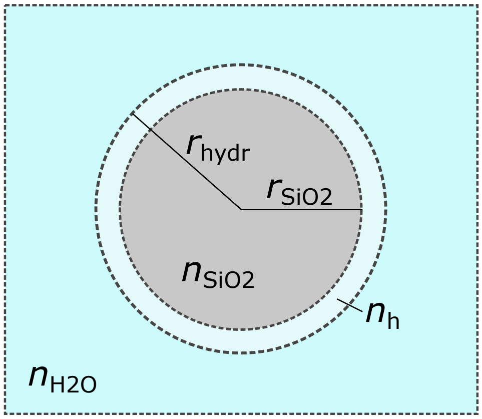

Due to negative surface charges, a hydration shell is formed when the SiO_2 nanospheres are dispersed in water. This leads to an increased effective radius ($r_{\mathrm{hydr}} > r_{\mathrm{SiO2}}$) as well as to an effective refractive index n_{eff}, which is lower than the one of the solid sphere n_{SiO2}. With our cavity sensor, we measure r_{hydr} and n_{eff}. The value for the intrinsic size r_{SiO2} can be obtained by scanning electron (sample A, see Supplementary Fig. 9) or transmission electron micrographs (sample B, see Supplementary Fig. 10). The value for the refractive indices of the bulk spheres are $n_{\mathrm{SiO2}} = 1.42$ (sample A, see Supplementary Fig. 9) and $n_{\mathrm{SiO2}} = 1.4537$ (sample B, see Supplementary Fig. 10). Following [1], we can calculate the mean refractive index n_{h} of the shell, by solving

$$\frac{n_{\mathrm{eff}}^2 - n_{\mathrm{H2O}}^2}{n_{\mathrm{eff}}^2 + 2n_{\mathrm{H2O}}^2} = \frac{(2n_{\mathrm{h}}^2 + n_{\mathrm{SiO2}}^2)(n_{\mathrm{h}}^2 - n_{\mathrm{H2O}}^2)r_{\mathrm{hydr}}^3 + (n_{\mathrm{SiO2}}^2 - n_{\mathrm{h}}^2)(2n_{\mathrm{h}}^2 + n_{\mathrm{H2O}}^2)r_{\mathrm{SiO2}}^3}{(2n_{\mathrm{h}}^2 + n_{\mathrm{SiO2}}^2)(2n_{\mathrm{H2O}}^2 + n_{\mathrm{h}}^2)r_{\mathrm{hydr}}^3 + 2(n_{\mathrm{SiO2}}^2 - n_{\mathrm{h}}^2)(n_{\mathrm{h}}^2 - n_{\mathrm{H2O}}^2)r_{\mathrm{SiO2}}^3}. \tag{3}$$

We get

$$n_{\mathrm{h}} = \sqrt{-\frac{p}{2} + \sqrt{\frac{p^2}{4} - q}}, \tag{4}$$

with

$$p = \frac{n_{\mathrm{eff}}^2(r_{\mathrm{SiO2}}^3 + 2r_{\mathrm{hydr}}^3) - n_{\mathrm{SiO2}}^2(2r_{\mathrm{SiO2}}^3 + r_{\mathrm{hydr}}^3)}{2(r_{\mathrm{SiO2}}^3 - r_{\mathrm{hydr}}^3)} \tag{5}$$

and

$$q = -\frac{n_{\mathrm{SiO2}}^2 n_{\mathrm{eff}}^2}{2} \quad . \tag{6}$$

Hence, the refractive indices of the hydration shell amount to $n_{\mathrm{h}} = 1.40 \pm 0.02$ for sample A and $n_{\mathrm{h}} = 1.39 \pm 0.04$ for sample B.

Supplementary Figure 12: Temporal evolution of the frequency shift histograms for the higher order transverse modes

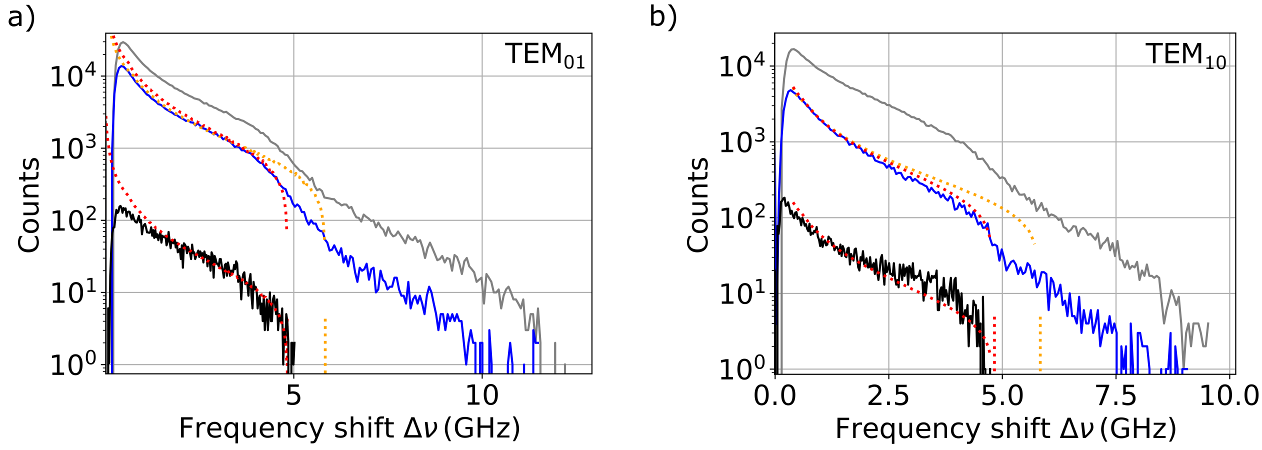

In a) and b) the time evolution of the measured frequency shift probability distributions for the higher order TEM_{01} and TEM_{10} modes are shown. The black solid line shows a single nanoparticle transit event, which is used for the reconstruction of the spatial coordinates of the nanoparticle track (main text Fig. 3b green and blue). It was measured within the first 2 hours. Dashed red line: simulated density of states for a single nanoparticle with refractive index n_{NP}=1.43 and r_{hydr}=77.4 nm, blue: frequency shifts of about 59 transits (after 2 hours measurement time) and gray: frequency shifts of about 210 additional transits (8 hours). In contrast to the time evolution of the TEM_{00} frequency shifts (see Fig. 2e, blue), the typical steep decay of the density of states for single nanoparticle transits doesn't occur for the first 2 hours measurement time for the higher order modes (Supplementary Fig. 11 a,b blue and simulated orange dashed line for r_{hydr}=82.3 nm). Since the TEM_{00} mode detects single nanoparticles with a low error probability, we can exclude agglomerations for the most transit events within the first 2 hours. Instead we assume a nanoparticle density inside the cavity which allows 2 separate nanoparticles being simultaneously inside the larger TEM_{01} and TEM_{10} mode at the same time. In future measurements, a higher probability for measuring only a single nanoparticle with all three modes could be achieved by reducing the concentration of the nanoparticle solution.

Supplementary Figure 13: Data evaluation for the TEM_{00}, TEM_{01} and TEM_{10} mode

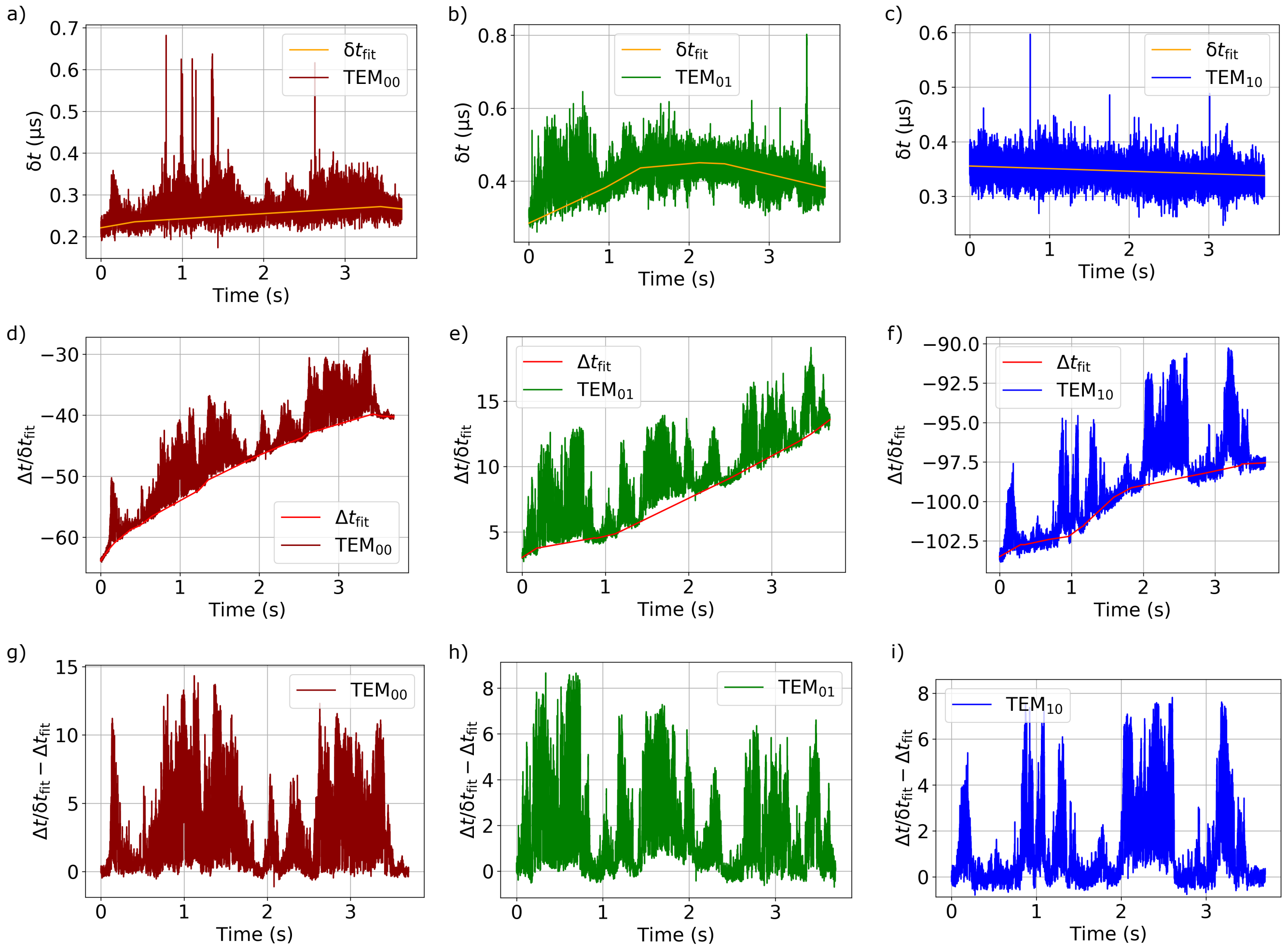

Here we show the measured raw data of the transit events, which is used for the calculation of the spatial coordinates of the SiO_2 nanoparticle with the size $r_{\mathrm{hydr}} = 75.3\,\mathrm{nm}$ (see Fig. 3b). In a), b) and c) the measured resonance linewidths of the TEM_{00}, the TEM_{01} and the TEM_{10} mode are depicted. d), e) and f) show the measured timeshifts, which are already recalculated in timeshift per linewidth units. g), h) and i) show the lineshift/linewidth with subtracted drift. More detailed information for the evaluation are given in Supplementary Fig. 5.

Supplementary Figure 14: Noise measurement for the TEM_{00}, TEM_{01} and TEM_{10} mode

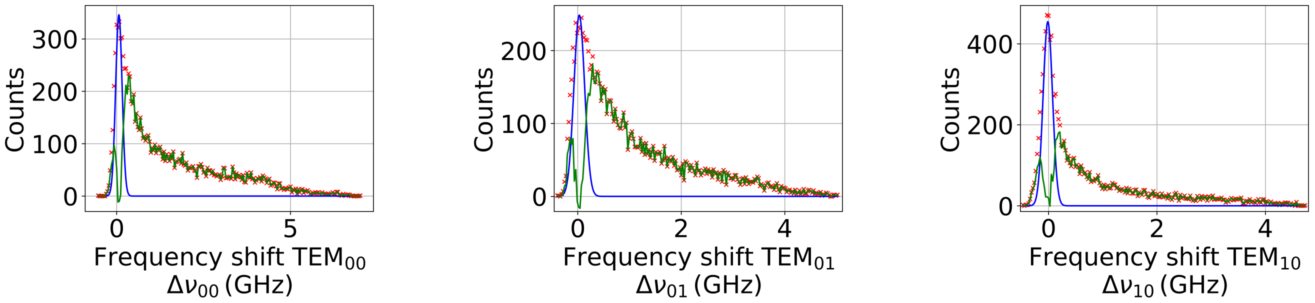

In order to deduce the noise form the measured frequency shifts in Fig. 3b, we plot the frequency shifts in an histogram. As already explained in Supplementary Fig. 8, the Gaussian fit to the measured counts at small frequency shift values gives the measurement noise: $\sigma_{00} = 0.13\,\mathrm{GHz}$, $\sigma_{01} = 0.15\,\mathrm{GHz}$ and $\sigma_{10} = 0.12\,\mathrm{GHz}$.

Supplementary Figure 15: Correlated TEM_{00} and higher order mode frequency shifts

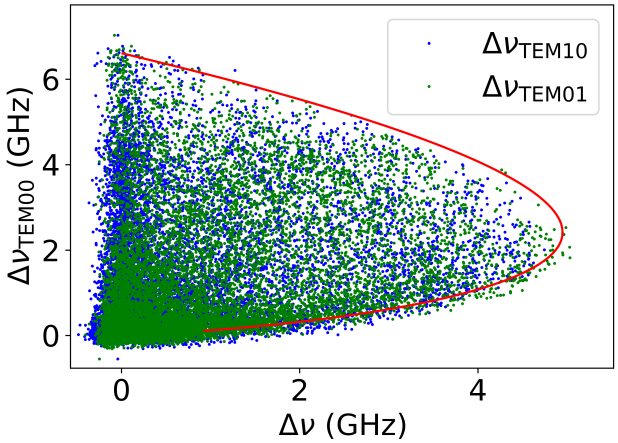

Here we show the correlation between the measured frequency shift of the fundamental mode $\Delta\nu_{\mathrm{TEM00}}$ and the higher order modes $\Delta\nu_{\mathrm{TEM01}}$ ($\Delta\nu_{\mathrm{TEM10}}$). When considering particle locations for maximal coupling e.g. on the x-axis (i.e. $y = z = 0$), we can calculate the corresponding frequency shifts ($\Delta\nu_{\mathrm{TEM00}}$, $\Delta\nu_{\mathrm{TEM10}}$), see the red line in Fig. 14. This yields the maximally observable shift pairs ($\Delta\nu_{\mathrm{TEM00}}$, $\Delta\nu_{\mathrm{TEM10}}$) since particle displacements along x, z will reduce one or possibly both values. The density of position states leads to a larger probability to find small shift values and only very few datapoints close to the top part of the red contour line, in agreement with the data. The observed correlation confirms the validity of using the frequency shifts of the two modes for position determination.

Supplementary Figure 16: Simulated Brownian motion and verification of the tracking algorithm

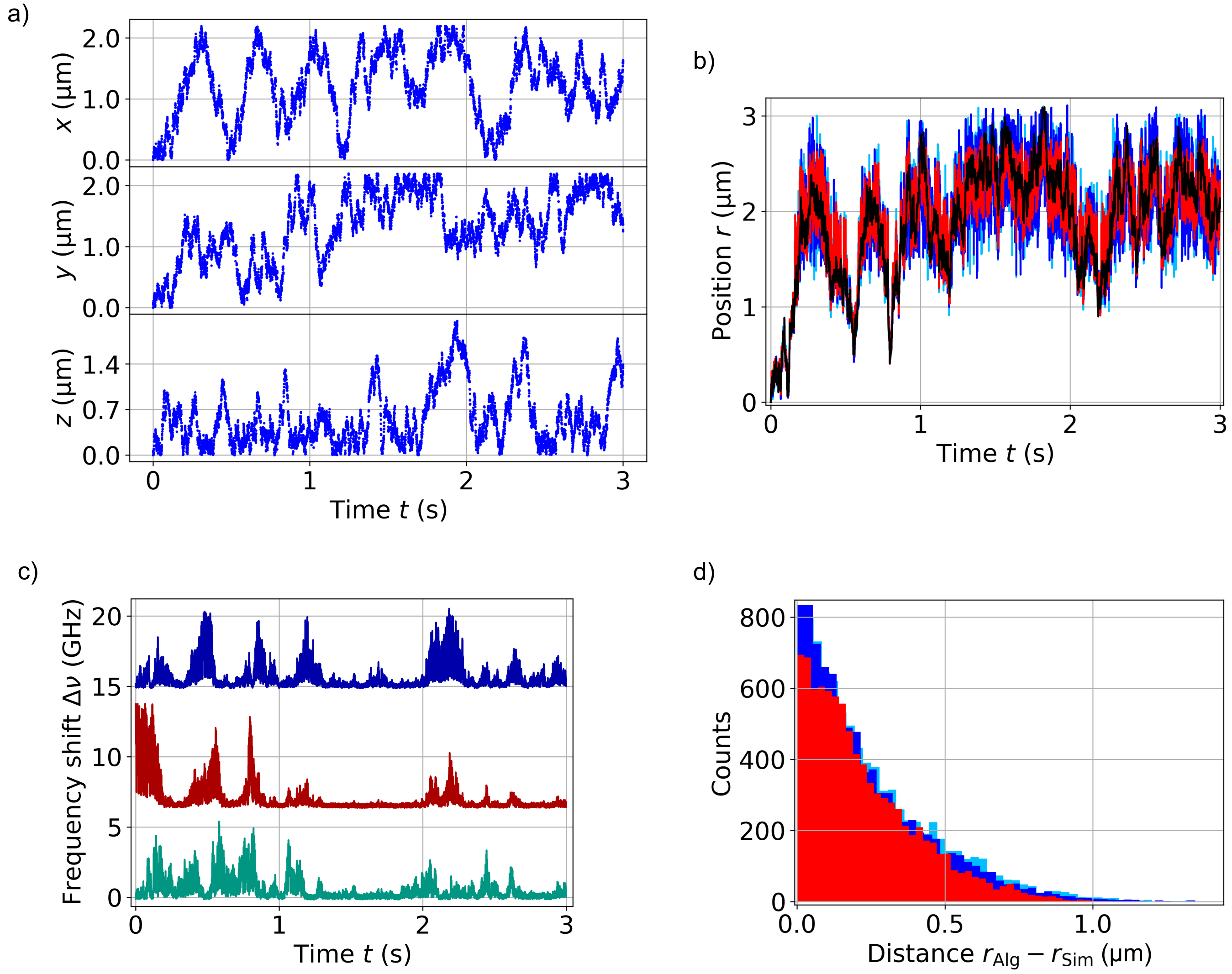

Due to measurement noise (see Supplementary Figure 14), each frequency shift triple at time t (see Fig. 3b) allows different possible positions of the nanoparticle inside the sensing octant. In the following we show that choosing the mean position fits best to the real track instead of choosing the minimal or the mean distance between two consecutive time steps. In order to verify this, we simulate different three-dimensional Brownian tracks with $dt = 1/(3\,\mathrm{kHz})$ time steps and the same theoretical diffusivity $D = 2.84\,\mu\mathrm{m}^2/\mathrm{s}$, of the SiO_2 nanoparticles with $r_{\mathrm{hydr}} = 75.3\,\mathrm{nm}$ size. In order to increase the time of the transit event, we fold each simulated track back into the sensing octant, when the sensing borders are reached

in x- and y-direction. Such a possible track is shown in a) and the absolute positions $r = \sqrt{x^2 + y^2 + z^2}$ is depicted in b) (black line).
In order to compare the simulated track and the track after our tracking algorithm, we perform two steps in a row. First, we calculate the frequency shifts from the simulated track and second we deduce the track from the frequency shifts. In the first step, we calculate for each position the frequency shift of the TEM_{00} (red), the TEM_{01} (green) and the TEM_{10} mode (blue), which would be produced by a SiO_2 nanoparticle and add the same measurement noise as in Supplementary Figure 14 to the shifts. The result is shown in c). In the second step, we calculate at each time t the possible positions of the nanoparticle. Here we take the mean position of all positions compatible with the measurement noise (red), the shortest distance (dark blue), as well as the mean distance (light blue) to the position before. All tracks are shown in b) together with the simulated track (black). Each track follows the simulated track, but small differences are visible. In d) the absolute distance to the simulated track is depicted in an histogram plot. The distribution is the narrowest for the mean position (red) and widest for the mean distance (light blue). Therefore we use the algorithm for the mean position in order to deduce the track of the nanoparticle from our measured frequency shifts.

Supplementary Figure 17: Mean squared displacement

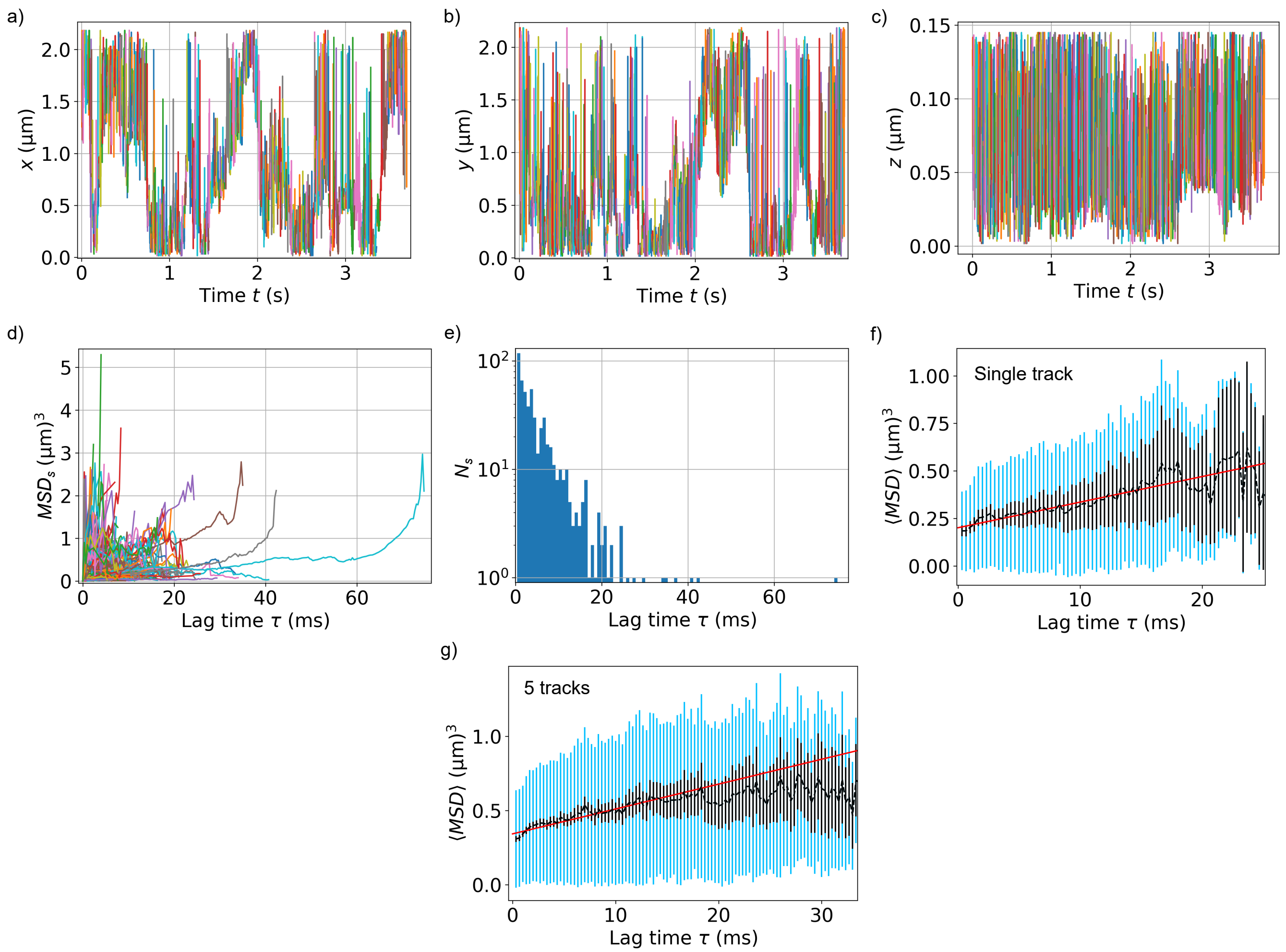

From the spatial coordinates in Fig. 3d, the mean squared displacement (MSD) is deduced. Here we divide the movement in sequences: A new sequence i starts if one of the spatial coordinates x, y, z reaches a border of the sensing octant. There, the determined position is folded back into the octant, such that the absolute distance to earlier positions becomes meaningless, and therefore a new sequence starts. a), b) and c) show the division of $x(t)$, $y(t)$ and $z(t)$ into different sequences by using different colors. For each sequence s and each sequence length N_s, the three-dimensional MSD is calculated with the following definition [2]

$$MSD_s(\tau) = \frac{1}{N_s - k} \sum_{t=0}^{N_s - k} (\mathbf{r}_s(t + k \cdot dt) - \mathbf{r}_s(t))^2, \quad \text{with } k = 1, 2, .., N_s. \tag{7}$$

Here, the lag time τ is defined as $\tau = \tau(k) = k \cdot dt$, with $dt = 1/f$ and $\mathbf{r}$ is $\mathbf{r} = (x_s, y_s, z_s)$. In our measurement, the scanning frequency of the cavity length is

$f = 3\,\text{kHz}$. All MSD segments are plotted in d) with different colors. The starting time for each sequence is set to zero. We derive the mean MSD $\langle MSD(\tau)\rangle$ by calculating the mean value at each lag time τ_k from all sequences $MSD_s(\tau_k)$:

$$\langle MSD(\tau)\rangle = \frac{1}{N(\tau)} \sum_s MSD_s(\tau) \tag{8}$$

Here, $N(\tau_k)$ is the number of $MSD_s(\tau_k)$ values at time τ_k. Since the length of the MSD segments MSD_s are different (shown in e)), also $N(\tau_k)$ is different to the overall number of segments. One can see that the nanoparticle reaches the sensing volume border within less than 10ms, which is dominated by the diffusion along z where the available distance is only $\lambda/(4n_\mathrm{m})$. f) shows $\langle MSD(\tau)\rangle$ as a function of τ as well as the standard deviations σ_i as error bars in blue and the statistical errors $10 \times \Delta x_i$ as error bars in black. We observe a linear increase of $MSD(\tau)$, which is in agreement with three-dimensional Brownian motion for the nanoparticle. Hence, a linear fit to $\langle MSD(\tau)\rangle$ (red line) gives the diffusivity D of the nanoparticle:

$$D = \frac{1}{6}\frac{d}{d\tau}\langle MSD(\tau)\rangle. \tag{9}$$

Only for small τ, a good statistics is available (see length distribution of the MSD_s segments in e)). In order to determine the optimal number of data x_ON for the linear regression, we follow [3] and get $x_\mathrm{ON} = 45$ or rather $\tau = 15\,\text{ms}$ for the last taken fitting point. We also weight the linear fit with the weighting function

$$w(\tau) = \frac{1}{\Delta x(\tau)}, \tag{10}$$

with

$$\Delta x(\tau) = \frac{\frac{1}{N(\tau)}\sum_s^{N(\tau)} \sigma_s(\tau)}{\sqrt{N(\tau)}} \tag{11}$$

and the standard deviation $\sigma_s(\tau)$ for the $MSD_s(\tau)$ value of segment s at lag time τ. As a result, we get $D = (2.2 \pm 0.3)\,\mu\text{m}^2/\text{s}$. This gives a hydrodynamic radius of $r_\mathrm{hydr} = k_\mathrm{B}T/(6\pi\eta\langle D\rangle) = (95.7 \pm 14.6)\,\text{nm}$. The specified uncertainty of the diffusivity stems from the standard error of the slope of the linear regression and the error for the hydrodynamic radius was calculated by Gaussian error propagation.

A better statistics is achieved when the MSD_s segments of several nanoparticle tracks are summed up. We used the data of five different tracks (including the single track shown in Fig. 3f) with an overall time duration of $t = 17.5\,\mathrm{s}$. As a result, we get the $\langle MSD \rangle$ shown in g). From the linear regression to the first $x_{\mathrm{ON}} = 62$ data points ($\tau = 20.7\,\mathrm{ms}$) we can deduce the diffusivity $D = (2.8 \pm 0.4)\,\mu\mathrm{m}^2/\mathrm{s}$ and the hydrodynamic radius $r_{\mathrm{hydr}} = (76.9 \pm 10.0)\,\mathrm{nm}$.

Supplementary Note 1: Cavity parameters for the different measurements

For our measurements we used two different kind of cavities: The cavity system shown in Fig. 1a (cavity 1) and the cavity shown in Supplementary Fig. 2 (cavity 2). In the following, the parameters of each cavity are specified and the results shown in the main article are related to the cavity systems used. In cavity 1, we measured SiO_2-spheres with hydrodynamic radius $r_{\mathrm{hydr}} = 71.3\,\mathrm{nm}$ (see Supplementary fig. 8) and in cavity 2 we measured SiO_2-spheres with hydrodynamic radius $r_{\mathrm{hydr}} = 75.3\,\mathrm{nm}$ (see Supplementary Fig. 9).

	**Cavity 1**	**Cavity 2**
Finesse TEM_{00}	18400	56710
Finesse TEM_{01}	-	47789
Finesse TEM_{10}	-	45668
Cavity length (µm)	27.3	5.4
FWHM TEM_{00} (MHz)	299	490
FWHM TEM_{01} (MHz)	-	581
FWHM TEM_{10} (MHz)	-	607
SM Fiber: $r_{c,x}$ (µm)	62.4	46.1
SM Fiber: $r_{c,y}$ (µm)	56.2	53.1
MM Fiber: $r_{c,x}$ (µm)	58.9	79.2
MM Fiber: $r_{c,y}$ (µm)	131.0	87.8
SM Fiber transmission (ppm)	17.6	17.6
MM Fiber transmission (ppm)	77	17.6
Fig. 2a		×
Fig. 2c	×	
Fig. 2d	×	
Fig. 2e		×
Fig. 3b		×
Fig. 3c		×
Fig. 3d		×
Fig. 3e		×
Fig. 3f		×

FWHM is the linewidth of the respective cavity mode. The data shown in Fig. 1b and 3a were recorded by cavity 1 with slightly different cavity parameters (different

cavity length, alignment) to those listed above.

Supplementary Note 2: Theoretical expected resonance amplitude decreases and frequency shifts

Depending on the nanoparticle position inside the cavity light field, different resonance amplitude decreases and frequency shifts occur. Both values reach their maximum value when the nanoparticle center is located at the antinode of the standing wave cavity light field. If the center is located at the node, a non-zero frequency shift occurs. This is due to the fact, that the nanoparticle hydrodynamical diameter is not negligible in comparison to the z-dimension of the antinode $\lambda/2n_{\mathrm{m}} = 293\,\mathrm{nm}$. Therefore, we have to consider a weighted polarizability of the nanoparticle [4], which is calculated by integrating over the spherical nanoparticle volume:

$$\langle\alpha\rangle = \frac{\alpha}{V_{\mathrm{NP}}}\int_{V_{\mathrm{NP}}} dV_{\mathrm{NP}} \left(\frac{w_0}{w(z)}\right)^2 \exp^{\frac{-2(x^2+y^2)}{w(z)^2}} \cos^2(kz), \tag{12}$$

with the polarizability for a sphere

$$\alpha = 4\pi r^3 \epsilon_0 \frac{n_{\mathrm{NP}}^2 - n_{\mathrm{m}}^2}{n_{\mathrm{NP}}^2 + 2n_{\mathrm{m}}^2}, \tag{13}$$

and using

$$w(z) = \frac{w_0}{\sqrt{1+(z/z_0)^2}} \tag{14}$$

for the beam radius of the Gaussian light distribution and w_0 is the beam waist. The maximum frequency shift is then given by

$$\Delta\nu_{\max} = \frac{\langle\alpha\rangle c}{2\lambda_{\mathrm{m}}\epsilon_0 V_{\mathrm{mode}}}. \tag{15}$$

Here, λ_{m} is the laser wavelength in medium, c speed of light in vacuum, and $V_{\mathrm{mode}} = \pi w_0^2 d/4$ is the cavity mode volume.

For the simulation in Fig. 2b we assume a linear change in frequency shifts from 0 to $\Delta\nu_{\max}$. The correlated resonance amplitude decreases can be calculated by the additional loss L_{NP} originating from light scattering and absorption by the nanoparticle.

At first, we consider an empty cavity, though filled with water, but no nanoparticle

is disturbing the signal. The transmission through this cavity is determined by the losses L_1, L_2 and the transmissions T_1, T_2 of the two cavity mirrors and the water loss L_m:

$$T_0 = \frac{4T_1T_2}{(L_1 + L_2 + T_1 + T_2 + 2L_\mathrm{m})^2}. \tag{16}$$

When the nanoparticle enters the light field, it produces an additional loss L_NP. Hence, the transmission can be expressed as

$$T(L_\mathrm{NP}) = \frac{4T_1T_2}{(2L_\mathrm{NP} + L_1 + L_2 + T_1 + T_2 + 2L_\mathrm{m})^2}. \tag{17}$$

The amount of loss L_NP depends on the nanoparticle position inside the light field and reaches its maximum at the antinode, with

$$L_\mathrm{p,max} = \frac{4\sigma_\mathrm{ext}}{\pi w_0^2} \tag{18}$$

and σ_ext being the extinction cross section of the nanoparticle and w_0 being the waist of the cavity light field. The extinction cross section depends on the size and refractive index of the nanoparticle and is taken from Mie Theory. For the nanoparticle losses L_NP we assume a linear change between 0 and $L_\mathrm{p,max}$. The theoretical decrease of amplitude ΔT depending on the losses L_NP produced by the nanoparticle can then be calculated as following:

$$\Delta T(L_\mathrm{NP}) = 100\% \left(1 - \frac{T(L_\mathrm{NP})}{T_0}\right). \tag{19}$$

Supplementary Note 3: Density of states - Theoretical frequency shift probability distribution for a single nanoparticle

Here we explain how we calculate the frequency shift probability distribution of the TEM_{00} mode, which we use to fit the data and thereby deduce the refractive index (see Supplementary Fig. 8).

Depending on the position (x_0, y_0, z_0) of the nanoparticle inside the TEM_{00} mode, an according frequency shift $\Delta\nu(x_0, y_0, z_0)$ occurs:

$$\Delta\nu(x_0, y_0, z_0) = \frac{\langle\alpha\rangle_{00} \cdot c}{2\lambda_{\mathrm{m}}\epsilon_0 V_{\mathrm{mode}}}, \tag{20}$$

with the weighted polarizability

$$\langle\alpha(x_0, y_0, z_0)\rangle_{00} = \frac{\alpha(n_{\mathrm{NP}})}{V_{\mathrm{NP}}} \int_{V_{\mathrm{NP}}} dV_{\mathrm{NP}} \cdot I_{\mathrm{Gauss}} \cos^2(k(z - z_0)) \tag{21}$$

and the Gaussian intensity distribution

$$I_{\mathrm{Gauss}} = \left(\frac{w_0}{w(z - z_0)}\right)^2 \exp^{\frac{-2((x-x_0)^2+(y-y_0)^2)}{w(z-z_0)^2}}. \tag{22}$$

Here, $k = 2\pi n_{\mathrm{H20}}/\lambda_{780}$ is the wavenumber depending on the wavelength in water $\lambda_{780}/n_{\mathrm{H2O}}$. For a given refractive index n_{NP}, the theoretical frequency shift probability distribution can be calculated and subsequently the derived frequency shift values plotted in an histogram.

Supplementary Note 4: Theoretical frequency shift matrices for the calculation of the 3D track

In Supplementary Note 3 we already introduced the calculation of the theoretical frequency shift matrix of the fundamental mode. Equation (20) (Supplementary Note 3) can be also used for the calculation of the TEM_{01} and TEM_{10} matrices, but the polarizabilities have to be adapted to

$$\langle\alpha(x_0, y_0, z_0)\rangle_{01} = \frac{\alpha(n_{\mathrm{NP}})}{V_{\mathrm{NP}}} \int_{V_{\mathrm{NP}}} dV_{\mathrm{NP}} \cdot \frac{8(x-x_0)^2}{w_{01}(z-z_0)^2} I_{\mathrm{Gauss}} \cos^2(k_{01}(z-z_0)) \tag{23}$$

for the TEM_{01} mode and

$$\langle\alpha(x_0, y_0, z_0)\rangle_{10} = \frac{\alpha(n_{\mathrm{NP}})}{V_{\mathrm{NP}}} \int_{V_{\mathrm{NP}}} dV_{\mathrm{NP}} \cdot \frac{8(y-y_0)^2}{w_{10}(z-z_0)^2} I_{\mathrm{Gauss}} \cos^2(k_{10}(z-z_0)) \tag{24}$$

for the TEM_{10} mode. In addition, we have to change the wavenumber to $k_{01} = k_{10} = 2\pi n_{\mathrm{H2O}}/\lambda_{785}$, since we probe the higher modes at a different wavelength (see Supplementary Fig. 1).

In order to deduce the spatial coordinates of the nanoparticle from the measured frequency shifts, the latter are compared with the theoretical frequency shift values. For the theoretical shift matrices we choose spatial pixel steps with 16.9 nm in lateral directions (x,y) and 1.6 nm in longitudinal direction (z). A suitable pixel size can be estimated from the measured frequency noise of each mode (see Supplementary Fig. 13). By including the measurement noise to the measured frequency shifts, we find multiple possible positions of the nanoparticle inside the theoretical frequency shift matrices at measurement time t_k. As a tracking algorithm we take from the multiple possible position the one which is closest to the earlier position at time t_{k-1}.

BIBLIOGRAPHY

[1] Y. T.C. Ko, J. P. Huang, and K. W. Yu. The dielectric behaviour of single-shell spherical cells with a dielectric anisotropy in the shell. *Journal of Physics Condensed Matter*, 16(3):499–509, 2004.

[2] Vincent Tejedor, Olivier Bénichou, Raphael Voituriez, Ralf Jungmann, Friedrich Simmel, Christine Selhuber-Unkel, Lene B. Oddershede, and Ralf Metzler. Quantitative analysis of single particle trajectories: Mean maximal excursion method. *Biophysical Journal*, 98(7):1364–1372, 2010.

[3] Xavier Michalet. Mean square displacement analysis of single-particle trajectories with localization error: Brownian motion in an isotropic medium. *Physical Review E - Statistical, Nonlinear, and Soft Matter Physics*, 82(4):1–13, 2010.

[4] A. A.P. Trichet, P. R. Dolan, D. James, G. M. Hughes, C. Vallance, and J. M. Smith. Nanoparticle Trapping and Characterization Using Open Microcavities. *Nano Letters*, 16(10):6172–6177, 2016.
